# Gallie technique in the treatment of odontoid fracture in pediatric: A case report

**DOI:** 10.1016/j.ijscr.2025.111175

**Published:** 2025-03-18

**Authors:** Yudha Mathan Sakti, T. Arief Dian, Karisa Kartika Sukotjo, Fuad Dheni Musthofa, Saeful Anwar, Pandhu Al Afghani Harlan

**Affiliations:** a Staff of Department of Orthopaedic and Traumatology, Faculty of Medicine and Nursing, Yogyakarta, Indonesia; b Resident of Department of Orthopaedic and Traumatology, Faculty of Medicine and Nursing, Yogyakarta, Indonesia; c Staff of RS PHC, Surabaya, Indonesia

**Keywords:** Pediatric cervical spine trauma, Modified Gallie procedure, Anterior cord syndrome

## Abstract

**Introduction:**

Pediatric cervical spine injuries (CSI) account for 60–80 % of all pediatric spine injuries and remains a challenge due to the difference in spinal anatomy compared to adults. There are currently no standardized method of diagnosis and treatment for CSI in children, hence identification and management remain challenging for orthopaedic surgeons.

**Case presentation:**

a 11-year-old girl present with weakness on right upper and lower limb due to hyperflexion injury after her father accidentally fell on her neck. Initial radiology workup showed normal bony alignments and led to the misdiagnosis of Guillain-Barre Syndrome. However, further CT and MRI workup revealed odontoid fracture compromising the spinal canal, which caused anterior cord syndrome. She was managed by Modified Gallie procedure, which involves passing a sublaminar wire in C1-C2 and using the resected part of C2 from the subsequent decompression as an onlay type graft. Her condition was stable post-operatively and 3-months follow up showed improved motor and sensory function in ASIA Score. Patient had even returned to school and regained mobilization using a walker.

**Discussion:**

Upper cervical injuries in younger patients are common due to their anatomy and flexibility allowing immediate self-reduction post dislocation, which may cause the initial normal radiography. Modified Gallie procedure and decompression was selected as the treatment to alleviate the need of additional bone graft.

**Conclusion:**

Cervical spine injury in children is a rare but challenging entity, where successful management requires an understanding of pediatric anatomical differences and the specific patterns of pathologies that frequently occur.

## Highlights

• There are currently no standardized method of diagnosis and treatment for CSI in children, hence identification and management remain challenging for orthopaedic surgeons
• Management of odontoid fracture of our patient include stabilization of the vertebral segments using Modified Gallie procedure, followed by decompression by hemilaminectomy and hemilaminotomy to address the spinal cord edema

## 1 Introduction

Although pediatric cervical spine injuries (CSI) are rare, with only 2 % of patients sustaining CSI, these cases constitute 60–80 % of all pediatric vertebral injuries [1]. The mortality rate of CSI accounts for 4 %, but the potential biopsychosocial impacts of such injuries on the individual, family, and broader society can be significant. The distinct anatomy and biomechanics between adult and pediatric spine result in different pattern and prognosis of injury. Key anatomical elements include ligamentous laxity, underdeveloped uncinate process, and shallow facet joint leading to its inherent instability and risk of injury. There are currently no standardized method of diagnosis and treatment for CSI in children, hence identification and management remain challenging for orthopaedic surgeons [2,3]. We present a case of odontoid fracture of C2 with spinal cord injury in a child, from initial misdiagnosis due to the radiography workup before reaching its definitive treatment.

## 2 Presentation of case

Three days prior to admission, a 11-year-old girl complained about pain on the upper back her neck after her father accidentally fell on her neck during play. No specific pain location was noted, but the patient reported no radiating pain to her extremities. The pain was accompanied with weakness on the right upper and right lower limbs immediately after the accident. Although mobility on all extremities was preserved, there was a decrease in strength observed specifically on the right side, along with numbness and tingling on her right leg. There were no further complaints and she was still able to control her micturition and defecation. The patient was immediately brought to a local private hospital, where she was treated by a pediatrician and underwent Brain CT scan with normal result. Her injury was then managed as Guillain-Barre Syndrome (GBS) and received antibiotic, anticonvulsant, and neuroprotectant as medication. However, her condition did not improve after 2 days and she was finally referred to a tertiary hospital for further treatment. There was no remarkable past medical history.

**Fig. 1:**
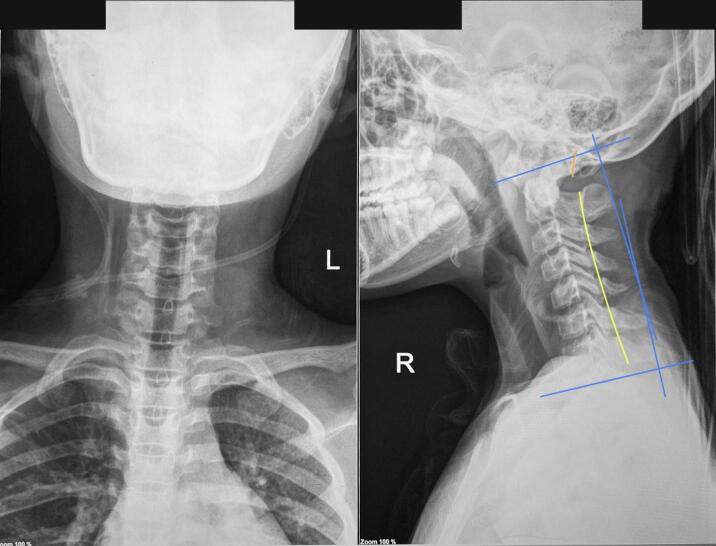
Cervical X-Ray in AP and Lateral view on the patient showing normal cervical anatomy without fracture nor listhesis.

**Fig. 2:**
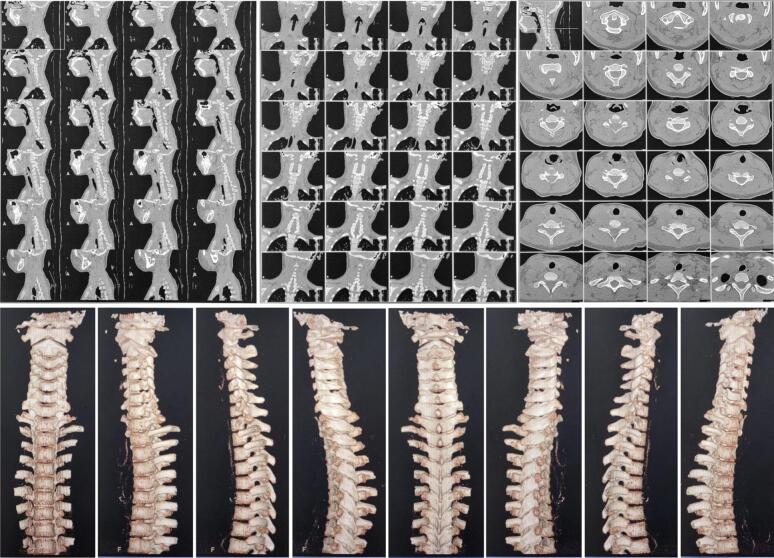
MSCT Cervical in axial, coronal, and sagittal view without contrast on our patient revealed retrolisthesis of odontoid, resulting in spinal canal stenosis on level VC1–2.

**Fig. 3:**
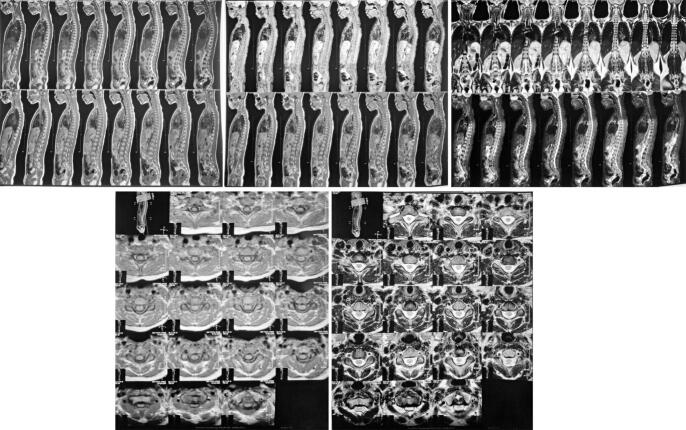
MRI Cervical-Thoracal-Lumbal-Sacral on axial, coronal, and sagittal view confirmed retrolisthesis of odontoid and severe canal stenosis along with spinal cord edema on level VC2.

No visible deformities were noted upon physical examination, but there was a midline tenderness on the back of her upper neck and paraspinal muscle spasm on the right side. ASIA Score showed decreased both sensory and motor function on the right side, but reflexes and autonomic function were within normal. Plain radiography did not show any abnormalities (Fig. 1). However, CT-Scan revealed retrolisthesis of C2, causing canal stenosis on level VC1–2 (Fig. 2). MRI confirmed severe spinal stenosis on C2 level with spinal cord edema (Fig. 3).

**Fig. 4:**
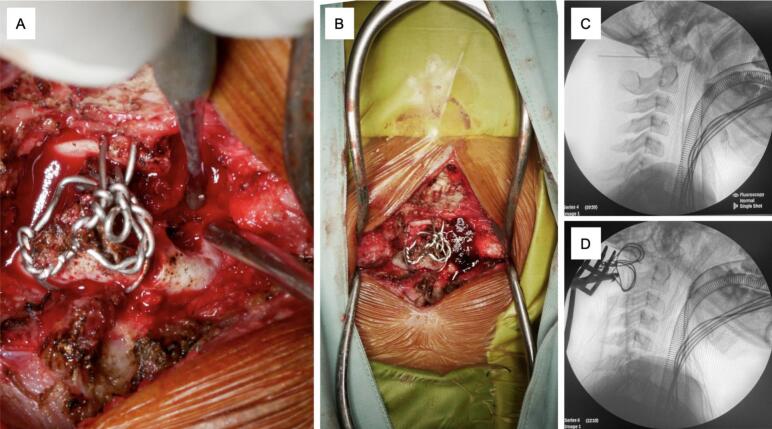
Hemilaminectomy, decompression, and Gallie Procedure. **A)** C1 Hemilaminectomy and C2 Laminotomy, **B)** Bone graft was inserted between C1 and C2, **C)** Pre-operative C-arm, **D)** Post-operative C-arm.

Patient was finally diagnosed with incomplete spinal cord injury at the level of C2 AIS C with suspected anterior cord syndrome and odontoid fracture on C2 spine Anderson D'Alonzo type II. She then underwent posterior C1-C2 fusion using Modified Gallie Procedure and decompression. Patient was put in prone position and the exact location of the incision is identified using a C-arm (Fig. 4c,d). The dissection is carried down the midline through the subcutaneous tissue and the fascia to the tips of the spinous processes to decrease bleeding. The vertebra level was confirmed intra-operatively with fluoroscopy as soon as bony structures are visible, which was done prior to any further dissection. Self-retaining retractors are used to maintain tension on soft tissues during exposure. The paraspinal muscles are elevated subperiosteally from the underlying laminae, using a Cobb elevator. Dissection is performed along the spinous processes, lamina, and lateral mass (bilaterally), which functions to minimize bleeding and muscle damage. After we identified the fracture site, two 1.0 mm SS-wires were inserted from the inferior posterior arch of C1 and the superior posterior arch of C2. Both wires were then secured to ensure symmetrical position of external occipital protuberance dan the spinous process. After stabilization was achieved, decompression was done by hemilaminectomy of the right C1 and hemilaminotomy of the right C2 (Fig. 4a), which resected axis was utilized as the onlay bone graft and inserted between arch of C1 and lamina of the C2 (Fig. 4b). The graft was secured by sublaminar wires and the wound was closed. These procedures were done by an orthopaedic surgeon specializing in spine surgery.

Her condition was stable postoperatively and immobilization was done using soft collar neck measured from the angle of the mandible up to the jugular notch for 12 weeks. Upon 3 months follow-up, ASIA Score showed both motor and sensory improvement. Patient no longer complained of numbness, weak right-hand grip has returned, and mobilization was recovered with assistance from a walker. The parent also reported that the patient had returned to school to continue her studies.

Informed consent from the patient was inquired prior to submission of this study. This case report has been reported in line with the SCARE Guideline [4].

## 3 Discussion

Although cervical spine trauma in children is relatively infrequent, the potential significant biopsychosocial impact from this injury highlights the importance of early identification and management. Epidemiology showed different characteristics of injury in different age groups due to interplay of anatomical and behavioral changes as the child ages. While most cervical injury was caused by blunt trauma due to motor vehicle accidents, especially in young adults, injury by falls were also a common cause observed in younger children as found in this case [2].

Our patient sustained upper cervical injury, which was a common pattern seen in younger children due to the greater relative size of the head compared to the rest of the body. This places the fulcrum of the cervical spine at the upper cervical region, increasing the likelihood of injuries in this area. The head is proportionately smaller as the child grows, shifting the fulcrum of the cervical spine downward, resulting individuals in these age groups more susceptible to lower cervical spine injuries [1]. Difference in elastic properties of the connective tissue in children also predisposes them to ligamentous injury as the connective tissue were able to stretch farther without fully-developed neck musculature. Underdeveloped uncinate processes from C3 to T1 also increases the risk of spondylolisthesis as the structure functions to limit lateral flexion. Rigid supporting muscles and ossified vertebral bodies seen in older children might offer superior protection against SCI, but at the cost of bony injuries [2].

Clinical presentation of motor weakness with sensation of numbness and tingling led to the diagnosis of anterior spinal cord injury. Generally, traumatic spinal cord injury is rare in children and incomplete SCI accounts for 75 % of cases [2]. Anterior cord syndrome typically manifests as motor symptoms along with loss of pain, non-discriminative touch and temperature perception. This type of injury commonly occurs due to anterior spinal artery occlusion and demyelination, which may result from non-traumatic etiologies [5].

Initial cervical X-Ray in this case did not reveal any abnormalities and had led to misdiagnosis of GBS. However, further imaging uncovered the presence of odontoid retrolisthesis, which compromise the spinal canal and resulted in anterior cord syndrome. This phenomenon of normal radiograph may be explained by the flexibility of pediatric spine caused by the shallow and horizontal facet joints. This anatomy allows immediate self-reduction following dislocation, resulting in seemingly normal bone alignment [2]. Hence, physicians must be wary of SCI presence despite normal radiographic exam when risk factors present. Risk stratification of SCI in adult patients used the five indicators of NEXUS (National Emergency XRadiology Utilization Study): focal neurologic deficit, midline spinal tenderness, altered level of consciousness, intoxication, and distracting injury. Meanwhile, Pediatric Emergency Care Applied Research Network (PECARN) criteria is developed to address CSI in blunt trauma for patients less than 18 years old using altered mental status, focal neurologic findings, neck pain, torticollis, substantial torso injury, conditions predisposing to cervical spine injury, diving, and high-risk motor vehicle crash [6]. Patients less than 3 years old were recommended to utilize Pieretti-VanMarcke Score, which uses Glasgow Coma Score, pattern of injury, and age as method of assessment [7]. However, cervical spine radiography remains as the appropriate methods of initial imaging modality due to less stochastic effects of radiation exposure and avoidable need of sedation [3].

**Fig. 5:**
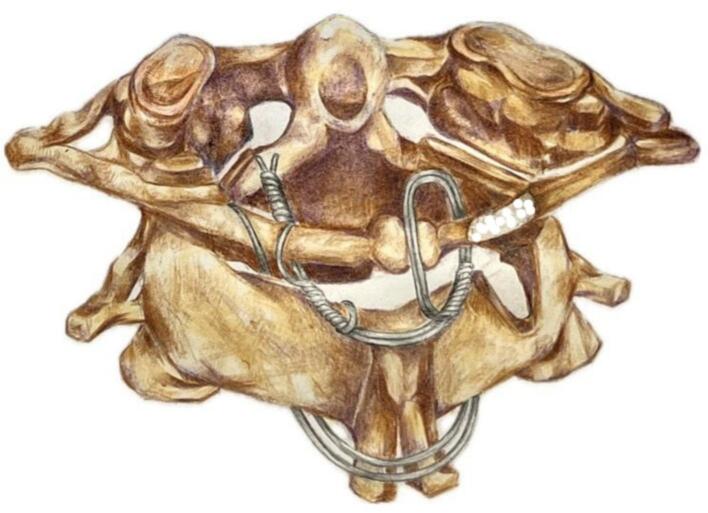
Illustration of the modified Gallie technique in our case, which involves passing a sublaminar wire in C1-C2 and using the resected part of C2 from the subsequent decompression as an onlay type graft.

Management of odontoid fracture of our patient include stabilization of the vertebral segments using the Gallie procedure, followed by decompression by hemilaminectomy and hemilaminotomy to address the spinal cord edema (Fig. 5). To alleviate the need of additional bone grafting, resected segment of previous decompression was utilized. Operative treatment was indicated as Type II odontoid fractures by with the Anderson-D'Alonzo classification often leads to atlantoaxial dislocation [8]. Common option for surgical fixation includes anterior percutaneous screw fixation, which has shown promising outcomes for management of odontoid fractures in adult patients. However, placing anterior standard screws in children can pose risks due to the considerable forces exerted during screw placement and has been associated with screw loosening, penetration, migration, or even severe neurovascular damage [9]. Therefore, Gallie procedure was selected due to the younger age of our patient correlates with smaller pedicle size, which may be unsuitable for screw placements. Gallie technique has also long been proven to provide solid fusion and stabilization in Type II odontoid fractures. According to previous studies, Gallie procedure provides less surgery time, less medical expense, and reduce intraoperative bleeding when compared with screw constructs [8,10]. This procedure had been reported in pediatric patients with satisfactory result and minimal limitations of neck rotation postoperatively [11]. Patient responded well to the treatment and was stable postoperatively. During 2 weeks of follow up, both motor and sensory function from the right extremities have improved as indicated by the ASIA scores. To the author's knowledge, the combination of decompression and Gallie procedure such as this case has not yet been reported before.

Our case report highlights the challenge in managing pediatric cervical traumas, as initial workup may be misleading and inability to identify possible cervical fracture may delay the definitive treatment. However, better SCI prognosis may be reached if potential risk factors for SCI are determined. Further studies are necessary to elucidate the standardized method of diagnosis and management of pediatric cervical trauma.

## 4 Conclusion

In conclusion, cervical spine injury in children is a rare but challenging entity, where missed diagnosis may lead to devastating consequences. Successful management requires an understanding of pediatric anatomical differences and the specific patterns of pathologies that frequently occur. Many controversies remain and high-quality evidence is required to determine best practice to provide better prognosis in pediatric CSI.

## Author contribution

Sakti, YM: Conceptualization, Supervision.

Anwar, S: Conceptualization, Supervision.

Yasser: Conceptualization, Reviewing, Editing.

Sukotjo, KK: Conceptualization, Reviewing, Editing.

Harlan, PAA: Writing, Reviewing, Editing.

Musthofa, FD: Writing, Reviewing, Editing.

## Consent

A copy of the written consent is available for review by the Editor-in-Chief of this journal on request.

## Ethical approval

The institutional review board of Sardjito General Hospital does not provide an ethical approval in the form of case report/case series.

## Guarantor

Yudha Mathan Sakti

## Source of funding

This research did not receive any specific grant from funding agencies in the public, commercial, or not-for-profit sectors.

## Declaration of competing interest

The authors report no declarations of interest.
